# Furin Targeted Drug Delivery for Treatment of Rhabdomyosarcoma in a Mouse Model

**DOI:** 10.1371/journal.pone.0010445

**Published:** 2010-05-03

**Authors:** Katarina Hajdin, Valentina D'Alessandro, Felix K. Niggli, Beat W. Schäfer, Michele Bernasconi

**Affiliations:** 1 Department of Oncology, University Children's Hospital Zurich, Zurich, Switzerland; 2 Experimental Infectious Diseases and Cancer Research, University Children's Hospital Zurich, Zurich, Switzerland; Bauer Research Foundation, United States of America

## Abstract

Rhabdomyosarcoma (RMS) is the most common soft tissue sarcoma in children. Improvement of treatment efficacy and decreased side effects through tumor-targeted drug delivery would be desirable. By panning with a phage-displayed cyclic random peptide library we selected a peptide with strong affinity for RMS *in vitro* and *in vivo*. The peptide minimal binding motif Arg-X-(Arg/Lys)(Arg/Lys) identified by alanine-scan, suggested the target receptor to be a proprotein convertase (PC). Expression profiling of all PCs in RMS biopsies and cell lines revealed consistent high expression levels for the membrane-bound furin and PC7. Direct binding of RMS-P3 peptide to furin was demonstrated by affinity chromatography and supported by activity and colocalization studies. Treatment of RMS in mice with doxorubicin coupled to the targeting peptide resulted in a two-fold increase in therapeutic efficacy compared to doxorubicin treatment alone. Our findings indicate surface-furin binding as novel mechanism for therapeutic cell penetration which needs to be further investigated. Furthermore, this work demonstrates that specific targeting of membrane-bound furin in tumors is possible for and suggests that RMS and other tumors might benefit from proprotein convertases targeted drug delivery.

## Introduction

Rhabdomyosarcoma (RMS), the most common soft tissue sarcoma in children, is thought to develop through disruption of muscle differentiation and can therefore arise anywhere in the body [1]. Treatment response and prognosis vary widely depending on location and histological RMS subtype.

Two main distinct histological subgroups exist with prognostic significance: embryonal rhabdomyosarcoma (eRMS) found in 60% and alveolar rhabdomyosarcoma (aRMS) found in 20% of patients. The latter represents the more aggressive subtype with the poorest prognosis at diagnosis [2]. The exact determination of the tumor mass and spread is important for tumor staging and appropriate therapy design consisting of the combination of surgery and chemotherapy. Since relapsing RMS are usually refractory to standard chemotherapy, the use of more aggressive therapies is needed with long lasting side-effects which affect normal child development.

Current therapies for RMS could be improved by targeting the tumor either by tumor specific compounds, or by delivering conventional drugs specifically to the tumor. No specific targeting approach is so far available neither for RMS nor any other pediatric embryonal tumor. Most work has focused on the development of monoclonal or single chain antibodies to tumor-associated antigens, coupled to an anticancer agent [3], [4], [5]. Unfortunately, such therapies showed limited efficacy in solid tumors such as RMS due to poor tumor penetration [6]. To overcome these drawbacks, small peptide-based compounds have been considered as carrier molecules to selectively deliver compounds to tumor specific receptors [7], [8]. Many promising lead compounds are being identified through screenings of large biological and synthetic peptide libraries [9], such as phage displayed random peptide libraries,[10], [11].

Proprotein convertases (PC) are a family of subtilisin-like serine proteases. They play a crucial role in the processing of various protein precursors ranging from hormones, growth factors, adhesion molecules, extracellular proteins to viral-envelop glycoproteins and bacterial exotoxins [12], [13]. Seven out of nine family members generate active proteins through post-translational endoproteolysis of precursors at the specific recognition sequence Arg-X(Arg/Lys)(Arg/Lys), namely furin, PC1/3, PC2, PC4, PACE4, PC5/6 A and B and PC7, whereof only furin, PC5B and PC7 are transmembrane proteins. They cycle between the surface and the trans Golgi network while processing their substrates [14], [15]. Endogenous expression of furin is detectable in normal tissue, while elevated furin expression is associated with several diseases including cancer [16], [17]. In particular, furin overexpression has been linked to aggressive metastatic tumors [18] presumably by activating cancer-promoting factors which include vascular endothelial growth factors (VEGF-C and D) [19], [20], transforming growth factor β (TGF-β) [21], insulin-like growth factor 1 receptor (IGF-R1) [22], bone morphogenic protein 4 (BMP-4) [23] membrane type 1 matrix metalloproteinase (MMP-1) [24] and several adamalysin metalloproteinases [25] as well as integrins [26]; or by inactivating tumor suppressors like Semaphorin-3B [27]. Inhibition of furin decreases tumor growth in some preclinical tumor models [28], [29], [30], while other tumor models indicate a more complex contribution of PCs to tumorigenesis [31], [32] Thus, although the dual role of PCs in tumorigenesis has to be further investigated, PCs represent a promising target to decrease or even prevent the activation of cancer-promoting factors [33].

We have previously identified two RMS targeting peptides through an *in vitro* screening of a phage-displayed random peptide library on RMS cell lines [34]. In this study, we improved the screening approach by combining *in vitro* and *in vivo* panning, and tested the identified RMS targeting peptide for selective drug delivery to RMS.

## Results

### Screening for phage-displayed peptides binding to RMS cells

To identify peptides that bind specifically to RMS, we performed *in vitro* and *in vivo* phage screening. First, a phage-displayed random cyclic peptide library was depleted of phage binding to normal cells by negative selection on either myoblasts (panning I) or fibroblasts (panning II). The precleared libraries were used in two parallel screenings on cultured RD cells for five rounds of selection. These yielded a phage pool that bound to RD cells 1200-fold for panning I and 900-fold for panning II over non-recombinant T7 (Supp. Fig. S1A and S1B). Subsequently, both *in vitro* selections were pooled and subjected to an *in vivo* screening on RMS xenografts. After two rounds of *in vivo* selection, phage-binding to tumors increased to 50-fold over non-recombinant T7 (Supp. Fig. S1C). To identify peptides displayed by the phages, 40 phages from each *in vitro* screening and 30 phages from the enriched population *in vivo* were randomly picked and sequenced. Analysis of the sequences revealed the recurrence of six phages (Table 1). Alignment of the peptide sequences indicated frequent presence of arginines and lysines, suggesting the importance of these basic residues. Four phages had the amino acid sequence Arg-X-Arg-Arg (RXRR), whereas X stands for a neutral, polar amino acid. Two phage clones (RMS-P3 and RMS-P6) had the sequence Arg-Thr-Lys-Lys (RTKK). RMS-P6 was the most abundant phage selected both *in vitro* as well as *in vivo* (43% and 27%, respectively).

**Table 1:** Alignment of phage displayed peptide sequences selected from RMS cells *in vitro* and *in vivo*.

Phage name	Displayed peptide sequence^A^	Frequency in both selections *in vitro*		Frequency in selection *in vivo*	
RMS-P6	CMGTTNT**R**T**KK**C	34/80	43%	6/30	27%
RMS-P3	CMGTINT**R**T**KK**C	14/80	18%	6/30	17%
RMS-P7	CGTGS**R**A**RR**SC	7/80	9%	3/30	10%
RMS-P10	CLTG**R**Q**RR**SSQC	4/80	5%	2/30	7%
RMS-P9	CSGPNV**R**S**RR**C	3/80	4%	2/30	7%
RMS-P8	CRTG**R**Q**RR**SSEC	3/80	4%	2/30	7%

A Phage-displayed consensus motifs are shown in bold letters.

### Selection and validation of RMS binding peptides

**Figure 1:**
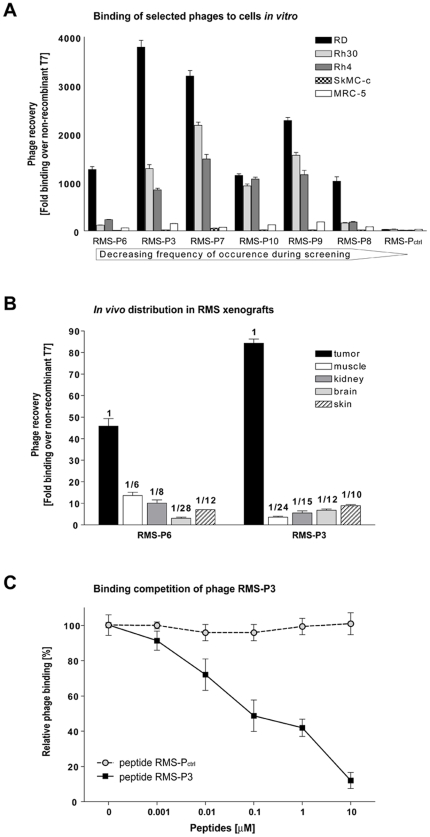
Validation of selected phages binding on RMS cells *in vivo* and *in vitro*. (A) Quantification of binding of single phage clones to different cell lines. Six phages selected from *in vitro* and *in vivo* screenings were validated individually in binding assays to three RMS cell lines (embryonal RMS: RD; alveolar RMS: Rh30 and Rh4) as well as to normal myoblasts (SkMC-c) and fibroblast cells (MRC-5). Phage clones are ordered according to the frequency of occurrence. Binding was calculated over non-recombinant T7, depicted are mean values ± SD of three independent experiments. (B) RMS-P3 and RMS-P6 phage distribution in RD mouse xenografts. Homing of phages to tumor was compared to control organs. Binding was normalized over phage T7 and tissue weight. Mean values ± SD of four independent experiments are shown. Numerical values for tumor to organ ratios are indicated above the bars. (C) Validation of RMS-P3 peptide sequence by competition of phage binding. 10^6^ RD cells were incubated for 30 min with increasing concentrations of RMS-P3 peptide (CMGTINTRTKKC) or control peptide (CSPNNTRRPNKC). Then, phages (10^9^ pfu) were added for 1 h. Phage binding was normalized over non-recombinant T7. Data are presented as percentage of maximal phage binding obtained in the absence of synthetic peptide. Error bars indicate mean ± SD from three independent experiments. Binding of phage RMS-P3 decreases significantly with increasing concentrations of synthetic peptide RMP-P3 (least-squares regression, N = 18, r^2^ = 0.889, b = −17.5, p<0.0001), but not for control peptide (N = 18, r^2^ = 0.0002, b = 0.055, p = 0.959).

The specificity of the enriched phages was tested in binding assays to RMS and normal cells individually (Fig. 1A). For each phage, binding was significantly better on cancer cells than on normal cells, except for control phage P_ctrl_. The most abundant phage, PMS-P6 (CMGTTNTRTKKC), bound 1270-fold to RD, 230-fold to Rh4 and 120-fold to Rh30 cells compared to the non-recombinant phage T7. Interestingly, phage RMS-P3 (CMGTINTRTKKC), differing from RMS-P6 only in one amino acid, bound 4 to 10 times better than phage RMS-P6 to all RMS cells tested, suggesting an important role for the isoleucine for binding efficiency. Phage RMS-P_ctrl_ (CSPNNTRRPNKC) arose spontaneously during the *in vitro* screening and had a similar sequence to RMS-P6 and RMS-P3 but showed no binding to tumor cells and was therefore used as negative control. Phages bound to the different RMS histosubtypes, alveolar and embryonal, equally well. Based on these results, phage RMS-P3 and RMS-P6 were further tested for their ability to bind to RMS tumors *in vivo* in mice bearing either eRMS (RD cells, Fig. 1B) or aRMS (Rh4 cells, Supp. Fig. S1D). Both phages bound significantly better to tumors than to any other organ (calculated with One-way ANOVA and Tukey-Kramer HSD). Tumor binding *in vivo* was up to 50-fold lower than *in vitro*. Enrichment of phages *in vivo* is usually much lower than *in vitro*. This is partly due to a higher background binding and to higher complexity of tissues, but it also might indicate that the target is less accessible or expressed at lower levels *in vivo* compared to cultured tumor cells. RMS-P3 revealed the best tumor/muscle ratio (24-fold) in both xenografted RMS histosubtypes and was therefore selected for further studies. Therefore, RMS-P3 cognate peptide was synthesized to test its ability to compete with phage RMS-P3 for the binding to RD cells *in vitro*. Constant amounts of phage RMS-P3 were incubated with increasing concentrations of peptide RMS-P3 or control peptide RMS-P_ctrl_ (Fig. 1C). We observed a significant correlation between increasing peptide concentrations and decreasing phage binding. Half-maximal inhibition (IC_50_) of phage binding by the synthetic peptide was approximately at 100 nM and the highest concentration tested (10 µM) decreased phage binding to 7.5%. Control peptide RMS-P_ctrl_ had no effect on phage RMS-P3 binding. We therefore conclude that RMS-P3 phage binding to RD cells is mediated by the displayed peptide and the synthetic peptide retains its binding specificity. The binding of phage RMS-P3 was further investigated on different cancer and normal cells (Supp. Fig. S1E). This experiment revealed a significant binding of RMS-P3 also to breast carcinoma, glioblastoma and melanoma cells compared to myoblasts and fibroblasts (One-way ANOVA with Tukey Kramer HSD test, p<0.05), suggesting that the potential target is overexpressed on different tumor cell lines.

### RMS-P3 peptide localizes on the cell surface and Golgi structures of tumor cells *in vitro* and *in vivo*

**Figure 2:**
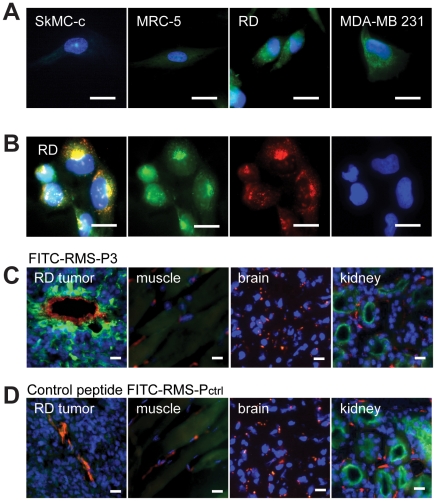
FITC-labeled RMS-P3 binding *in vitro* and distribution *in vivo*. (A) Visualization of FITC-RMS-P3 peptide binding in normal and cancer cells *in vitro*. Cultured myoblasts (SkMC-c), fibroblasts (MRC-5), RMS cells (RD) and breast carcinoma cells (MDA-MB 231) were incubated with 100 nM FITC-RMS-P3 peptide for 1 h at 37°C. Fluorescence microscopy of fixed cells is shown. (B) Living RD cells were incubated with FITC-RMS-P3 peptide, subsequently fixed and stained with giantin (red). Colocalization is shown in the merged picture by yellow. Blue staining (DAPI) indicates the nuclei. Magnifications: 40x, scale bars: 20 µm. (C) Distribution of FITC-RMS-P3 and control peptide FITC-RMS-P_ctrl_ in mice xenografts of RD cells. FITC-RMS-P3 or control FITC-RMS-P_ctrl_ peptide were injected i.v. and after 10 minutes of circulation, mice were perfused and tumor and control organs were collected. Cryosections were double-labeled with mouse endothelial vessels markers CD31 and MECA-32 (red). Nuclear staining by DAPI is shown in blue. Magnifications: 40x, scale bars: 20 µm.

To characterize the binding of peptide RMS-P3 on cultured cells, the corresponding fluorescein-conjugated peptide (FITC-RMS-P3) was incubated with myoblasts, fibroblasts, RMS or breast carcinoma cells. A bright fluorescence could be seen on cancer cells, but not on normal cells (Fig. 2A). Interestingly, the fluorescence did not exclusively localize on the cell surface, but seemed to accumulate inside the cells. To address the subcellular localization of RMS-P3 in RD cells, we compared the pattern of the fluorescent peptide with that of giantin, a marker for the Golgi compartment (Fig. 2B). A clear colocalization of FITC-RMS-P3 and the giantin staining was visible in reticular and perinuclear distribution, indicating that FITC-RMS-P3 binds both to the cell surface as well as to Golgi-associated structures. Confocal microscopy confirmed FITC-RMS-P3 internalization in RD cells (Supp. Fig. S2). Binding of FITC-RMS-P3 to RMS tumors was further investigated *in vivo* in RD xenografts. The labeled peptide was specifically detected within endothelial and peri-endothelial tumor cells whereas control organs like muscle and brain remained unaffected. Only kidneys showed a low peptide uptake (Fig. 2C). However, the control peptide FITC-RMS-P_ctrl_ - which did not accumulate in the tumor - showed a strong kidney uptake (Fig 2D). Remarkably, FITC-RMS-P3 was internalized in tumor cells, as shown by confocal microscopy (Supp. Fig. S2). In conclusion, FITC-RMS-P3 shows preferential accumulation in RMS tumors, confirming the tumor specificity *in vivo*.

### Identification of the RMS-P3 binding motif

**Figure 3:**
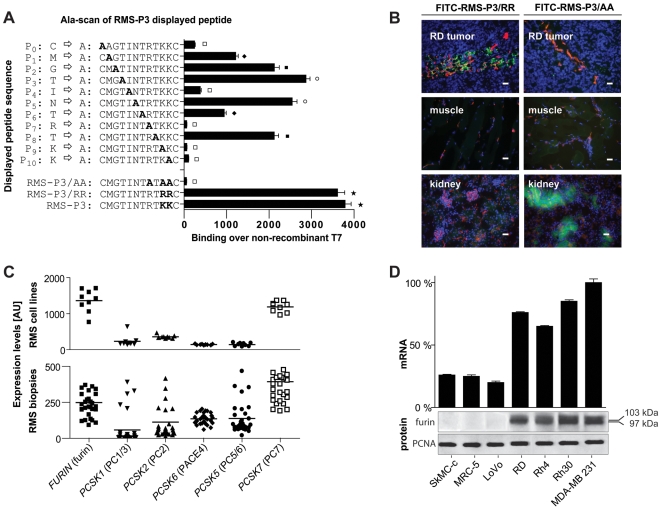
Identification of the receptor for RMS-P3. (A) Alanine-scan of the RMS-P3 sequence. RMS-P3 was mutated by replacing each amino acid residue with alanine (codon GCT). All phage mutants were tested individually for binding to RD cells *in vitro*. Mutated residues are indicated in bold. Phage binding was quantified over non-recombinant T7 and compared to the binding of wild type phage RMS-P3. Depicted are mean values ± SD of three independent experiments. Phage RMS-P3/RR shows no different binding to RD cells than phage RMS-P3, but both are significantly different from all other alanine mutants (One-way ANOVA, with Tukey Karmer HSD test, p<0.05). Bars not connected by same signs are significantly different from each other. (B) FITC-RMS-P3/RR and FITC-RMS-P3/AA distribution in RD xenograft mice. After tail vein injection of both FITC-RMS-P3/RR or control peptide RMS-P3/AA and circulation of 10 min, mice were perfused, tumors and control organs were dissected and FITC-peptide distribution (green) was evaluated in cryosections under fluorescence microscopy. Blood endothelial stainings (CD31 and MECA32) are shown in red, nuclear staining in blue (DAPI). Magnification: 20x, scale bar 50 µm. (C) Expression of proprotein convertases (PCs) measured by microarray profiling of 9 RMS cell lines and 30 RMS biopsy samples. Expression of PCs recognizing the cleavage site R(X)(R/K)(R/K) were considered. Each cell line or biopsy is represented by one data point. (D) Furin expression in different cell lines. Normal myoblasts (SkMC-c), fibroblasts (MRC-5), RMS cell lines (RD, Rh4, Rh30), furin-deficient colorectal carcinoma cells (LoVo) and furin-positive breast carcinoma cells (MBA-MB 231) were tested for furin expression by qRT-PCR (upper graph). SkmC-c and MRC-5 show both significantly lower RNA levels than any tumor cells (One-way ANOVA, Tukey Kramer HSD tests, p<0.05), but similar values to Lovo negative control (not significant). mRNA expression levels are depicted relative to the sample with the highest expression after normalization to GAPDH. Western blot (lower panels). Immature membrane-bound furin was detected at 103 kD, mature furin at 97 kD. PCNA was used as loading control.

To identify the minimal binding motif necessary for binding of RMS-P3, new phage clones were synthesized whereby each amino acid was singularly replaced by alanine (Ala-scan). All phage mutants were tested individually for their binding to RD cells (Fig. 3A). Comparing RMS-P3 mutants to RMS-P3, a dramatic decrease in binding could be observed when Arg at position 7 (60-fold decrease) or both Lys (57-fold decrease at position 9, 56-fold at position 10) were exchanged. Replacing Ile at position 4 had a minor but significant effect and resulted in 10-fold decreased binding. When all basic amino acids at positions 7, 9 and 10 were replaced by Ala (RMS-P3/AA), binding was almost completely abolished (70-fold decrease). To test whether the presence of any basic amino acid is sufficient for binding, both Lys were replaced by Arg (RMS-P3/RR). Results revealed no significant difference in binding to RD cells between RMS-P3 and RMS-P3/RR, indicating that any polar basic residues are sufficient for binding, but both are significantly different from all other alanine mutants (One-way ANOVA and Tukey Karmer HSD test, p<0.05). Since exchange of Lys to Arg decreases the hydrophilic character of the peptide and therefore lowers the kidney uptake *in vivo*, we tested the distribution of fluorescently-labeled peptide RMS-P3/RR *in vivo* and compared it to the Ala mutant RMS-P3/AA as negative control (Fig. 3B). Accumulation of FITC-RMS-P3/RR could be observed in RMS tumors, but not in control organs (muscle and kidney); the Ala mutant RMS-P3/AA did not accumulate in the tumor, but was found in the kidneys. These results confirm the specificity *in vivo*, and allowed us to continue our investigations with the peptide sequence RMS-P3/RR.

### Identification of proprotein convertases as candidate receptor

The minimal binding motif allowed a database search for proteins containing the sequence RX(R/K)(R/K) to deduce hypothetical target receptor(s). Indeed, this consensus sequence could be found in the premature, inactive proform of several proteins, which are processed by serine endoproteases, namely proprotein convertases (PCs). This led us to hypothesize that one of the seven PCs cleaving at the recognition site RX(R/K)(R/K) might be the binding target of RMS-P3/RR.

Supporting this notion, mRNA expression levels of all PCs with the recognition site RX(R/K)(R/K) were evaluated in 9 RMS cell lines and 30 RMS biopsy samples using Affymetrix gene expression profiles (Fig. 3C). In all tested cell lines and biopsies, levels of Furin and PC7 were significantly higher than other PCs (One-way ANOVA, Tukey Kramer HSD tests, p<0.05; Fig. 3C). Hence, either furin or PC7 were further considered as potential targets for RMS-P3/RR binding. Since furin has been linked to tumorigenesis before [18], [35], we concentrated our effort on this enzyme. Expression of furin was investigated at mRNA and protein level in myoblasts, fibroblasts and RMS cells. MDA-MB 231 [19] and LoVo [36], [37] cells were used as positive and negative controls, respectively. Furin expression was detected by qRT-PCR in all RMS cell lines at high levels, while myoblasts and fibroblasts showed low levels of furin transcripts comparable to the cell line LoVo (Fig. 3D, upper panel). Western blot detected expression of membrane-bound forms of furin (immature profurin at 103 kD, mature furin at 97kD) in RD, Rh4, Rh30 and MDA-MB 231 cells (Fig. 3D, lower panel). These results confirm high expression of furin in RMS cells supporting the hypothesis that furin might be the receptor for RMS-P3/RR binding.

### RMS-P3/RR binds to furin *in vitro*

**Figure 4:**
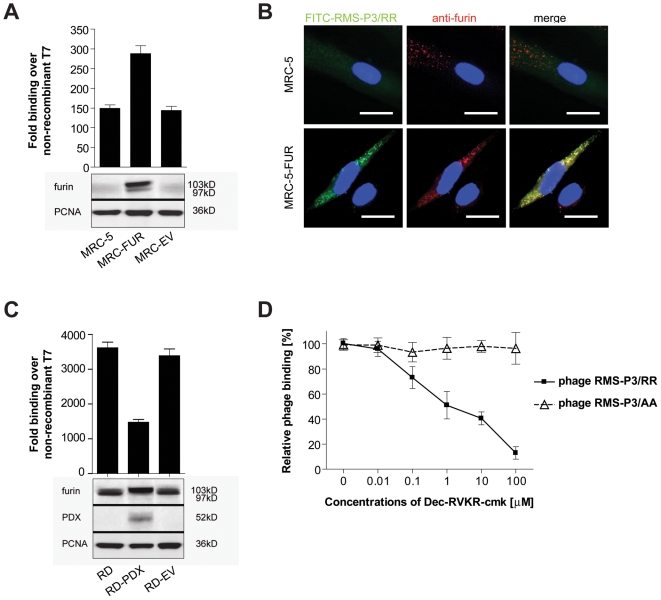
RMS-P3/RR phage binding correlates with furin expression levels. (A) Binding of phage RMS-P3/RR to untransfected (MRC-5), furin-overexpressing (MRC-FUR) and empty vector transfected MRC-5 fibroblasts (MRC-EV) was tested *in vitro*. Phage binding was quantified over control T7. Results of three independent experiments ± SD are shown. Western blot of the same samples for furin expression and PCNA as loading control (A, lower panels) (B) Micrographs of MRC-5 fibroblasts (upper row) and MRC-5-FUR (lower row) cells incubated with 100 nM FITC-RMS-P3/RR (green) for 1h at RT, fixed and stained with the anti-furin-antibody Mon-152 (red). Nuclei are visualized by DAPI staining (blue). Yellow indicates overlap of the two stainings. Magnification: 40x; scale bars: 20 µm. (C) Inhibition of furin maturation in α1-PDX-transfected RD cells reduces phage binding. Phage RMS-P3/RR binding to untransfected RD, furin-inhibited RD-PDX and empty-vector transfected RD-EV cells was determined over non-recombinant T7 in three independent experiments (means ± SD). Expression of the inhibitor α1-PDX was confirmed by western blotting using 100 µg cell extract with antiserum against α1AT (C, lower panels). (D) Effect of the specific PC-inhibitor Dec-RVKR-cmk on phage RMS-P3/RR binding. Competition was performed with increasing concentrations of Dec-RVKR-cmk and constant amounts of phage (10^9^ pfu). Phage binding was calculated over T7. RMS-P3/RR phage binding decreased with increasing concentrations of Dec-RVKR-cmk (least-squares regression, N = 18, r^2^ = 0.883, b = −17.9, p<0.0001), but binding of control phage RMS-P3/AA did not.

To provide direct evidence for binding of RMS-P3/RR to furin, we reasoned that furin-overexpression should result in increased phage RMS-P3/RR binding. Fibroblasts, expressing low levels of furin, were stably transfected with furin cDNA (MRC-5-FUR). Expression of immature and mature forms of furin was confirmed by Western blotting (Fig. 4A, lower panel), whereby the immature form was predominant. Phage RMS-P3/RR did bind significantly better to MRC-5-FUR than to MRC-5 or MRC-5-EV (pairwise comparisons with students t-tests: MRC-5-FUR vs. MRC-5: p = 0.0010, MRC-5-FUR vs. MRC-5-EV: p = 0.0008; Fig. 4A, upper panel). To confirm these results, untransfected and furin-transfected fibroblasts were incubated with FITC-RMS-P3/RR and furin expression was analyzed by immunofluorescence. MRC-5-FUR cells showed a strong peptide binding in contrast to untransfected cells. Moreover, a clear colocalization of furin and FITC-RMS-P3/RR was observed (Fig 4B).

On the other hand, inhibition of furin maturation should lead to a decreased binding of RMS-P3/RR. RD cells were stably transfected with α1-antitrypsin Portland (α1-PDX), a serpin, which inhibits irreversibly furin activity [38], and named RD-PDX. This led to the accumulation of unprocessed profurin, as indicated by the increased intensity of the 103kD band (Fig. 4C, lower panel). Phage RMS-P3/RR bound 2.5-fold less to RD-PDX cells as compared to empty-vector transfected RD cells (pairwise student's t-tests: RD-PDX vs. RD p = 0.0002, RD-PDX vs. RD-EV p = 0.0004 Fig. 4C, upper panel). This was further confirmed in competition experiments between the peptide-based irreversible inhibitor Dec-RVKR-cmk and phage RMS-P3/RR on RD cells (Fig. 4D). Increasing concentrations of Dec-RVKR-cmk resulted in decreased phage binding to RD cells. At 1 µM Dec-RVKR-cmk phage RMS-P3/RR binding was only half-maximal, whereas the unspecific binding of control phage RMS-P3/AA remained unaffected. These findings suggest that Dec-RVKR-cmk and phage RMS-P3/RR have the same binding site.

**Figure 5:**
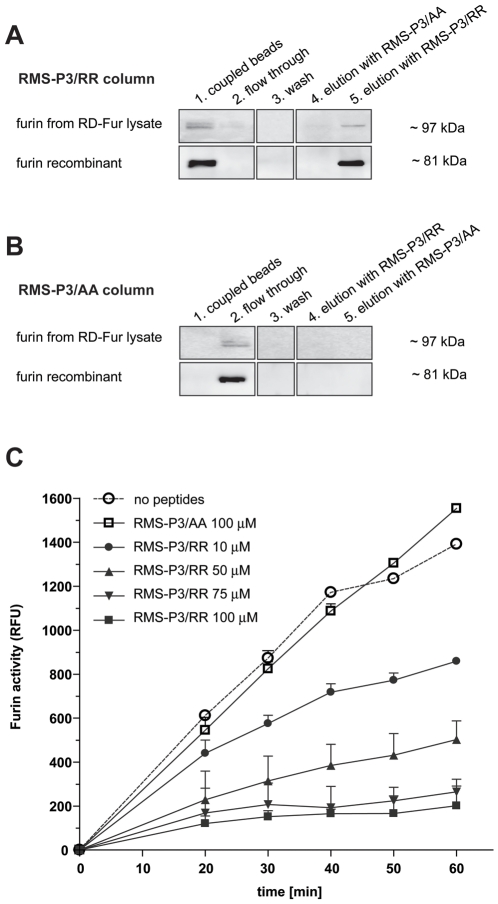
RMS-P3/RR peptide binds directly to furin and inhibits its activity. Lysates of RD-FUR cells (approximately 1 mg of total protein, upper rows) or purified recombinant furin (250 ng; lower rows) were loaded on a RMS-P3/RR column (A) or on the control peptide column RMS-P3/AA (B). The columns were washed 10 times to remove unbound furin. The loaded RMS-P3/RR-column was eluted first with control peptide RMS-P3/AA, then with peptide RMS-P3/RR. The RMS-P3/AA column was eluted first with RMS-P3/RR and subsequently cross-eluted with the control peptide RMS-P3/AA. The presence of furin in aliquots from the columns (1) after binding, (2) flow through, (3) last wash, (4) control elution and (5) competitive elution with RMS-P3/RR (in A) and RMS-P2/AA (in B), respectively, was detected by western blotting. (C) Fixed amounts of recombinant furin (3.3U) were incubated with increasing concentrations of RMS-P3/RR peptide or with control peptide (RMS-P3/AA). Furin activity was measured with the fluorogenic substrate Boc-RRVR-AMC at excitation 370 nm/emission 460 nm. The results of three independent experiments ± SD are shown.

To further demonstrate a direct interaction between furin and the synthetic peptide RMS-P3/RR, the peptide was coupled covalently to an NHS-activated sepharose column. Furin-transfected RD cells (RD-FUR) were lysed and the membrane fraction was incubated with the RMS-P3/RR-column. Unbound proteins were removed and bound proteins were eluted competitively with a concentrated RMS-P3/RR peptide solution. The washes from the column prior to specific elution showed no detectable furin. However, competitive eluates contained detectable amounts of membrane-bound furin (97kDa), indicating that the peptide can specifically dissociate furin from the column (Fig. 5A) which was not the case for the control peptide RMS-P3/AA. 5B). By incubation of the RMS-P3/RR column with recombinant human furin without membrane-domain (81 kD) and competitive elution with the peptide as above, Western blot analysis of the different fractions detected a furin band in the specific eluate (Fig. 5A). In contrast, a control RMS-P3/AA-column did not bind recombinant furin (Fig. 5B). These results strongly suggest that RMS-P3/RR peptide binds to mature furin, regardless whether it is in its membrane-bound or soluble form.

To test whether RMS-P3/RR can inhibit furin activity, we monitored the activity of furin with a fluorogenic substrate in the presence of increasing concentrations of RMS-P3/RR (Fig. 5C). Indeed, a concentration dependent decrease of furin activity was observed, and at a concentration of 100 µM RMS-P3/RR did reduce furin activity to 14% of the control. Slopes of all RMS-P3/RR peptide concentrations of at least 50 µM are significantly different from corresponding RMS-P3/AA peptide concentration and control (One-way ANCOVA with interaction effect time*peptide samples, 50 µM, 75 µM and 100 µM RMS-P3/RR peptide: p<0.0001; for 10 µM p = 0.241).

Taken together all these results demonstrate that RMS-P3/RR can directly interact with furin, and strongly suggest that the peptide binds to the membrane-bound and soluble form of mature furin at its active site.

### RMS-P3 binding correlates with furin expression *in vivo*

**Figure 6:**
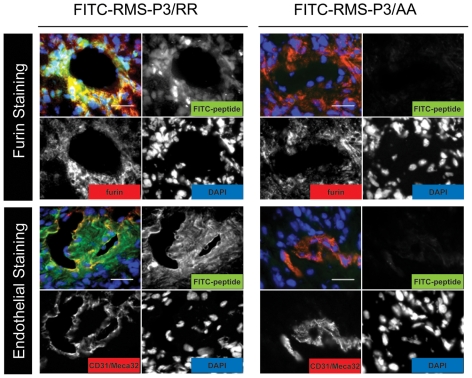
FITC-RMS-P3/RR and FITC-RMS-P3/AA distribution in mice bearing RD xenografts. Peptides were injected i.v. and after circulation the mice were perfused, tumors and control organs were removed and peptide distribution was evaluated on cryosections by fluorescence microscopy. Depicted are overlay images (B, upper left panel) and single channel pictures. FITC-peptide stainings (green), either furin or endothelial stainings (CD31, MECA32) in red and cell nuclei in blue (DAPI) are shown. Magnification: 40x, scale bars 50 µm.

To validate RMS-P3 binding to furin *in vivo*, we first tested the tumor homing ability of phage RMS-P3/RR to RD, RD-FUR and RD-PDX derived tumors. Phage RMS-P3/RR showed a 2-fold increased homing to RD-FUR compared to RD and a 45-fold increased binding compared to RD-PDX tumors. Binding to RD-FUR cells was significantly higher than to RD cells, and both were significantly higher than bindings to any other organ (Supp. Fig. S3). Moreover, localization of FITC-labeled RMS-P3/RR and RMS-P3/AA peptides after i.v. injection was evaluated in cryosections of RD xenografts (Fig. 6). Cryosections were stained either for furin (upper panels) or for the blood vascular markers CD31 and MECA-32 (lower panels). Around the blood vessels, strong peptide fluorescence was observed, indicating that the peptide penetrated about 30 µm into the tumor. No fluorescence was detectable after injection of the control peptide. Finally, furin colocalized with peptide RMS-P3/RR fluorescence around the blood vessels. The peptide accumulated in tumor cells. These results confirm homing of peptide RMS-P3/RR to RMS tumors *in vivo* and underscore binding to furin as potential intracellular targeting mechanism.

### Targeted delivery of doxorubicin reduces RMS tumor growth

**Figure 7:**
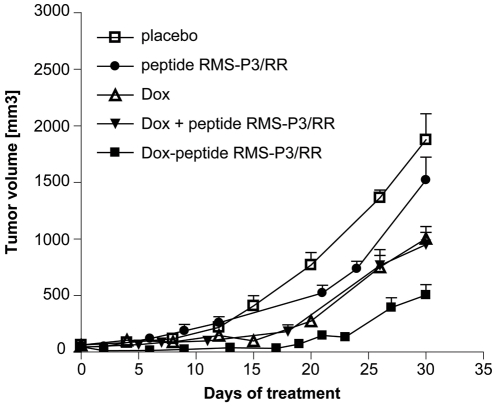
Targeted drug delivery with Dox-RMS-P3/RR in NOD/SCID mice bearing RD xenografts. Five groups of 6 mice were treated when tumors reached 60 mm^3^ in size. Treatment consisted of weekly tail vein injections for 30 days. Mice were treated with 10 µg/week of free doxorubicin (**▵**), 10 µg doxorubicin equivalent/week of doxorubicin-coupled RMS-P3/RR peptide (▪), with molar equivalent of free peptide RMS-P3/RR (•), 10 µg/week free doxorubicin plus molar equivalent of free peptide RMS-P3/RR (▾) or with vehicle alone (PBS, □). Treatment efficacy was best for Dox-RMS-P3/RR, significantly better than for Dox alone or other treatments. Pairwise student's t-tests on day 30, Dox-RMS-P3/RR vs. Dox alone: p = 0.042, Dox-RMS-P3/RR vs. peptide RMS-P3/RR: p = 0.0004, Dox-RMS-P3/RR vs. placebo: p<0.0001.

To investigate the potential of RMS-P3/RR for targeted drug delivery, doxorubicin was coupled to RMS-P3/RR (Dox-RMS-P3/RR). Mice with size-matched RMS xenografts were treated with Dox-RMS-P3/RR, doxorubicin and peptide RMS-P3/RR mixed together (Dox + RMS-P3/RR), doxorubicin alone (Dox), RMS-P3/RR alone, and vehicle alone (PBS). Targeting caused a significant delay in tumor growth compared to untargeted drug delivery (Fig. 7). Tumors were significantly smaller when treated with Dox-RMS-P3/RR than with Dox alone, Dox and peptide, peptide alone or vehicle (student's t-test, p<0.05). During therapy, no behavioral changes were observed that would indicate CNS toxicity, and after the therapy, organs were examined and no overt tissue damage was observed, i.e. no brain, heart or nephrotoxicity (data not shown). Hence, we conclude that targeted drug delivery by RMS-P3/RR to furin increases the treatment efficacy for RMS tumors.

## Discussion

Here, we report the isolation through phage display of a RMS targeting peptide and the identification of the proprotein convertase furin as its corresponding target receptor. The selected peptide showed promising therapeutic potential for targeted drug delivery in a pre-clinical RMS mouse model.

The majority of the phage-displayed peptides selected by a combination of *in vitro* and *in vivo* screening contained dibasic amino acids suggesting their importance for binding to RMS tumors. Two phage clones, RMS-P3 (CMGTINTRTKKC) and RMS-P6 (CMGTTNTRTKKC), differing only in one amino acid, showed strongest binding to RMS tumor cells. RMS-P3 was chosen for further studies because of its high affinity to RMS and the good tumor/organ ratios in both embryonal and alveolar histosubtypes *in vivo*. The corresponding synthetic RMS-P3 was validated by competition assays and a FITC-labeled RMS-P3 revealed accumulation in endothelial and peri-endothelial tumor cells but not in normal endothelial cells in RMS xenografts. Moreover, the peptide was able to penetrate into the cytoplasm and accumulate in the Golgi suggesting a possible receptor-mediated internalization.

Through single amino acid substitutions of the phage-displayed peptide sequence, it was possible to identify the minimal essential motif required for binding to RMS cells as Arg-X-(Arg/Lys)(Arg/Lys): lysines and arginines were interchangeable without loss of affinity towards RMS cells. This allowed us to design a peptide with decreased hydrophilic character and therefore less kidney-prone *in vivo* by replacement of lysines with arginines, RMS-P3/RR (CMGTINTRTRRC) retained the RMS targeting specificity *in vitro* and *in vivo*.

Several lines of evidence suggest that furin, the predominant member of the PC family, is indeed a target receptor. First, membrane-bound furin from cell lysates of furin-overexpressing RD cells binds to RMS-P3/RR as shown by affinity chromatography. Same results could be obtained with the soluble recombinant furin. Second, furin overexpression in fibroblasts increased binding of phage RMS-P3/RR. Third, staining of furin and FITC-RMS-P3/RR on furin-overexpressing fibroblasts evidenced a perfect colocalization in immunofluorescence. Fourth, phage RMS-P3/RR preferentially homed to furin-overexpressing RMS tumors and showed decreased binding to RMS tumors with inhibited furin activity. Fifth, phage RMS-P3/RR binding to RD cells was inhibited by the addition of the PC specific inhibitor Dec-RVKR-cmk. Sixth, RMS-P3/RR inhibited furin activity in a concentration dependent manner. Taken together, these results validate furin as a relevant target for RMS-P3/RR binding.

We do not rule out that other PCs, in particular PC7 which is also consistently expressed in RMS, are involved in the binding, or that additional mechanisms contribute to tumor binding and/or internalization. Interestingly, many of the homing peptides in the literature contain an R/KXXR/K, R/KXR/K, or R/KXR/KR/K motif, e.g. RGR [39], LyP-1 [40], RMS-I and RMS-II [34]. Recently, in an elegant series of works the group of Ruoslahti showed that the presence of an exposed R/KXXR/K motif at the C-terminus of a targeting peptide leads to cell, vascular, and tissue penetration through binding to neuropilin-1 (NRP-1) [41], [42]. Notably, this C-end rule (CendR) predicts that even internal R/KXXR/K motives can be exposed by proteolytic cleavage. These findings can be extended and suggest that proteolytical processing of tumor homing (poly)peptides is an important step for specific tumor targeting. In our system, binding of RMS-P3/RR to furin (or PC7) and on RMS cells might be a required intermediate step, but other molecules, such as NRP-1, might be involved in the downstream events. In addition to that, arginine-rich peptides can penetrate cells by various mechanisms [43], macropinocytosis being one. A peptide (CAYHRLRRC) very similar to RMS-P3/RR, targeting the macropinocytotic pathway, was selected by panning on leukemia and lymphoma cells [44]. Interestingly, both cell lines used for this study, MOLT-4 [45], [46] and K562 [47] express furin, or a protease with similar specificity. In this cellular background, furin or a related protease might be the unidentified receptor and play an important function in macropinocytosis.

These findings underline the potential importance of furin and other PCs for tumor targeting, but to validate RMS-P3/RR as tumor specific therapeutic target several points will have to be addressed. We have observed accumulation of the RMS-P3/RR peptide in tumors, particularly around blood vessels, where it colocalizes with furin, implying that tumor endothelial cells and the surrounding tissue might have increased furin expression at their surface, and therefore we conclude that RMS-P3/RR can target both human and mouse furin. It will be necessary to determine the biodistribution of a radiolabeled RMS-P3/RR to confirm the tumor specificity and accumulation and to test whether other tissues/conditions are involved in our pre-clinical model. Generally, furin is ubiquitously expressed and necessary for homeostasis of normal cells, raising concerns about the clinical translation of therapies based on activity inhibition. Furin is overexpressed in several cancers [18], [48], [49], however, only increased cell surface expression in tumors would allow specific targeted therapies with homing molecules. There are several examples of cellular proteins that are increased at the cell surface in tumors [50], and are receptors for homing peptides [51],[52]. Furin processing and transport to the cell surface might be specifically increased in tumor cells, as response to increased need to process cell matrix components required for tumor proliferation and tissue invasion. Therefore, the cancer-specific regulation of furin processing and transport to the cell surface needs to be further investigated to fully understand the mechanisms underlying RMS specific targeting by RMS-P3/RR and to allow improvement of furin-based tumor targeting approaches. Our results underline the importance of furin and PCs in tumor progression, and the need to develop novel approaches to target furin and PCs activity. Specific inhibitors of furin activity are being developed and improved [53], [54]. It will be important to test these inhibitors in pre-clinical settings, to verify their biodistribution, and better understand their clinical potential.

Peptide RMS-P3/RR conjugated with doxorubicin increased its therapeutic efficiency in RMS xenografts compared to free doxorubicin, and it could inhibit furin activity *in vitro*. The application of a targeting peptide which simultaneously inhibits furin and delivers drugs to the tumor site unveils interesting new therapeutic opportunities. Since doxorubicin is used as standard compound in the second-line treatment of RMS, our results indicate a therapeutic benefit with RMS-P3/RR as vector for a targeted drug delivery. Furthermore, when tested on different tumor cell lines phage RMS-P3 showed a very good binding to breast cancer, glioblastoma and melanoma. In conclusion, this study represents the first report on therapeutic targeting embryonal tumors. RMS-P3/RR-mediated drug delivery through furin might be therapeutically effective for RMS targeting and might be considered for other tumors as well.

## Materials and Methods

### Ethics Statement

All the animal experiments were approved and monitored by the Veterinary Office of the Canton of Zurich according to the Swiss Federal Law.

### Statistical analysis

Statistical analysis was performed using JMP statistical software (v7.0.2; SAS institute inc., Cary, NC). Data are expressed as mean±SD. Statistical significance was tested with unpaired two-tailed Student's *t*-tests, or for multiple comparisons analysis of variance (ANOVA) with Tukey Kramer HSD posthoc tests. To test for linear effects in time dependent experiments, we used linear least-squares regressions or ANCOVA. The differences were considered to be significant if *P*<0.05.

### Cell lines and cell culture

RD (eRMS), MRC-5 (fetal lung fibroblasts), MDA-MB-231 (breast adenocarcinoma) and LoVo (colorectal adenocarcinoma) cells were obtained from ATCC (LGC Promochem, France), SkMC-c and SkMC-p from PromoCell (Germany). Rh4, Rh18, Rh30 and Rh36 cells (aRMS) from P. Houghton (St. Jude Children's Research Hospital, Memphis, TN) and FLOH-1 (aRMS) from the Olga Hospital in Stuttgart (Germany). U87MG (glioblastoma) and A365 (melanoma) from the Department of Dermatology of the University Hospital Zurich (Switzerland). Ruch2 (eRMS, botryoid subtype) and Ruch3 (eRMS) were established in our laboratory [55]. All cells were maintained in high glucose DMEM supplemented with 10% fetal calf serum (Bioconcept, Switzerland), except for LoVo cells (Ham's F12 with 10% FCS) and myoblasts (Ham's F10 with 15% FCS and 2.5 ng/ml human basic fibroblast growth factor 2 (Sigma)) in 10% CO2 at 37°C. All media were from Gibco (Invitrogen, Switzerland) and contained 2 mM L-glutamine, 100 U/ml penicillin and 100 µg/ml streptomycin.

### Plasmids and transfections

pcDNA3.1(+) containing either full length furin (FUR) [56] or α1-AT Portland (α1-PDX), were generous gifts of Andres JP Klein-Szanto (Fox Chase Cancer Center, Philadelphia, PA). For stable transfections 5×10^6^ RD or MRC-5 cells were electroporated with 8 µg of the vectors pcDNA3.1(+)-FUR, pcDNA3.1(+)-α1-PDX or empty pcDNA3.1(+)-EV with the Nucleofector Kit R (Amaxa, Germany) using the program O-017. Cells were selected with 1 mg/ml G418 (Promega, Switzerland) in growth medium.

### Phage-displayed random peptide library production and screenings

Random peptide libraries were constructed in the lytic T7 phage as previously described [39], [57]. Oligonucleotides encoding random (NNK) cyclic peptides with the general structure CXnC (whereas X is any amino acid and n = 7 to 10) were cloned into the T7Select 415-1 vector arms according to the manufacturer's instructions (Novagen, Switzerland). A stop codon was inserted following the peptide coding sequence to avoid problems posed by concatamers ligations into the vector arms. The initial library diversity was evaluated to be 10^8^.

#### Negative selection:

10^6^ normal myoblasts (panning I) or fibroblasts (panning II) were detached with 2.5 mM EDTA-PBS and reconstituted in DMEM/1%BSA before overnight incubation with 10^9^ pfu of the phage library in 1 ml DMEM/1%BSA at 4°C. After cell sedimentation, phage were rescued from the supernatant by amplification in the E.coli strain BLT5403 (Novagen) and used for biopanning.

#### *In vitro* selection:

10^6^ RD cells were detached and, reconstituted as described above. Cells were incubated with 10^9^ pfu of the precleared library for 2 hours at 4°C under rotation. Unbound phages were removed by four washing steps with DMEM/1%BSA and cells were treated for 30 minutes with 1% NP-40 on ice. Bound phage were rescued and amplified. Phage enrichment was calculated after every round of panning over binding of non-recombinant T7 control phage.

#### *In vivo* selection:

Phage (10^10^ pfu) from *in vitro* biopannings were tail vein injected into mice bearing size-matched RMS tumors (500 mm^3^) as described in [57].

### Mutagenesis of phage displayed peptide sequences (Ala-scan)

Each codon of RMS-P3 was replaced by the alanine codon GCT. Oligonucleotides (Microsynth, Switzerland) were designed to have EcoRI and HinDIII sites and cloned into the T7Select 415-1 vector arms. Packaging and amplification in E.coli BLT5403 were performed following manufacturer's instructions (Novagen). Single phage clones were verified by DNA sequencing.

### Affimetrix gene expression profiling

mRNA expression levels of different proprotein convertases were evaluated from previously generated gene expression data of 30 RMS biopsy samples [58] and from newly acquired data of 9 RMS cell lines and 4 myoblasts cultures.

### Quantitative Real time PCR

Total RNA was extracted with the RNeasy Kit (Quiagen, Switzerland). Following DNase treatment, samples (1 µg) were reverse-transcribed with Oligo(dT) primers using the Omniscript Reverse Transcription Kit (Qiagen). qRT-PCR detection of furin and the house keeping gene GAPDH was performed with assay-on-demand Hs00965485_g1 and Hs99999905_m1, respectively (Applied Biosystems) and normalized to GAPDH. Experiments were performed in triplicates. Mean values and standard deviations were calculated based on the results of three biological replicates.

### Western blotting

10^7^ cells were denatured in RIPA buffer supplemented with 1 mM PMSF and Roche Complete Protease inhibitor (Roche, Switzerland) for 30 minutes on ice. Total cell extract (100 µg) was separated on 4–12% NuPAGE Bis-Tris gels (Invitrogen) and blotted on nitrocellulose membranes (Schleicher & Schuell, Germany). Blots were blocked with 3% BSA, incubated with the first antibody overnight at 4°C and with the corresponding HRP-conjugated secondary antibody for 1 hour at RT. Enhanced chemiluminescence detection system (SuperSignal West Femto, Pierce, Perbio Science, Switzerland) was used for detection of furin (MON-152, 1∶750; Alexis Corporation, Switzerland). Anti-α_1_-Antitrypsin antibody produced in rabbit (A0409; 1∶1000, Sigma-Aldrich), PCNA (1∶1000, Bioscience, Switzerland).

### Synthesis of peptides

The cyclic peptides CSPNNTRRPNKC (RMS-P_ctrl_), CMGTINTRTKKC (RMS-P3), CMGTINTRTRRC (RMS-P3/RR) and CMGTINTATAAC (RMS-P3/AA) were synthesized using standard FMOC chemistry in a solid-phase synthesizer by Eurogentec (Belgium). Fluorescein (FITC)-conjugated peptides contained a spacer of two glycines at the amino-terminus of the cysteine. Doxorubicin-succinyl-GG-CMGTINTRTRRC (Dox-RSM-P3/RR) was synthesized by BiomerTechnology (Hayward, CA) in a purity of >90%.

### Competition assays

10^6^ RD cells were detached (2.5 mM EDTA-PBS) and reconstituted (DMEM/1% BSA). Cells were preincubated with 100 µl of peptides (RMS-P3 or RMS-P_ctrl_) or general PC inhibitor decanoyl-Arg-Val-Lys-Arg-chloromethylketone (Dec-RVKR-cmk, Calbiochem, Switzerland) for 30 minutes. Phages (10^9^ pfu) were added for 1 hour at RT under continuous rotation. Cells were washed four times with DMEM/1% BSA; titer of bound phage was determined by titration with BLT5403 and calculated over non-recombinant T7.

### Furin activity

The enzymatic activity of furin was determined by the cleavage of the fluorogenic substrate Boc-RRVR-AMC (Alexis biochemicals) in presence or absence of peptides. Human recombinant furin (3.3U for reaction; Alexis biochemicals) was preincubated for 15 minutes at RT with the peptide RMS-P3/RR or the peptide RMS-P3/AA as negative control at increasing concentrations. For each assay, the same concentration of substrate (100 µM) was added to a solution containing 50 mM Tris HCl with 1 mM CaCl_2_ (pH 7) in a total volume of 100 µl. The reaction was followed for 1h while measuring the fluorescence every 10 minutes in a spectrofluorometer at excitation 370 nm/emission 460 nm.

### RMS-P3/RR affinity chromatography

RMS-P3/RR or control peptide RMS-P3/AA were dissolved in 0.2 M phosphate buffer pH 7.2 to 1 mg/ml and covalently linked to NHS-activated sepharose matrix SpinTrapTM (GE Healthcare, Amersham Biosciences, Switzerland). Free residual active groups were blocked according to manufacturer's instruction. Peptide columns were incubated either with 400 µl RD-FUR cell lysate in non-denaturating RIPA buffer (approximately 1 mg of total protein) or with 5U of recombinant human furin (approximately 250 ng of pure enzyme, Alexis Corporation) in 100 µl TBS (pH 7.5) overnight at 4°C under continuous rotation. After washings with 10 column volumes of TBS, each peptide (2 mg/ml in TBS) was applied to the column for 2 hours at 4°C. Competitive elution from the RMS-P3/RR column was performed first with the control peptide RMS-P3/AA then with RMS-P3/RR, each in two volumes of column buffer. The column with the control peptide RMS-P3/AA was eluted first with RMS-P3/RR followed by an elution with RMS-P3/AA.

### Fluorescence microscopy

5×10^4^ cells grown in chamber slides (BD Biosciences, Switzerland) were incubated for 1 hour at 37°C either with 100 nM FITC-RMS-P3 or FITC-RMS-P_ctrl_. Then, cells were washed with Dulbecco's PBS (Amimed, Bioconcept, Switzerland), fixed with 4% PFA for 15 minutes and permeabilized with 1% Triton X-100 in PBS for 15 minutes at RT and stained with Mon-152 (1∶100, Alexis Corporation). Polyclonal anti-giantin antibody (1∶1000, Abcam, Cambridge, UK). AlexaFluor594-labeled IgG antibodies (Invitrogen) were diluted 1∶300 in PBS/0.1%BSA. Cells were stained with 4′,6-diamidino-2-phanylindole (DAPI), washed twice with PBS and mounted with Vectashield Mounting medium (Reactolab SA, Switzerland). All images were obtained on an Axioskop 2 mot plus Fluorescent microscope (Carl Zeiss Visions, Switzerland).

### Animal models

CD1-Nu/nu mice (4 to 6 weeks old, Charles River, Germany) were used for the *in vivo* screening, and 6 weeks old NOD/SCID IL2R γ-/- mice were used for all other studies. RMS cells (5×10^6^ in 150 µl HBSS) were injected subcutaneously into the dorsolateral flank of the mice. Both diameters (d) of the spherical tumors were measured twice weekly and the volume was calculated with the general formula V = (4/3) πr^3^, whereby r = (d_1_+d_2_)/4. Prior to the experiment, mice were anesthetized with 0.015 ml/g avertin by i.p. injection. 1 mg/ml of FITC-peptide in 250 µl PBS was injected by tail vein injection into mice bearing tumors 150 mm^3^ in size. After 10 minutes circulation, mice were perfused, tumor and control organs were dissected and frozen in O.C.T. embedding medium (Leica Microsystems, Germany). 10 µm cryosections were washed with PBS, fixed for 15 minutes in ice-cold methanol and air-dried. Endothelial stainings were performed using rat monoclonal antibodies against mouse markers CD31 and MECA-32, both at the dilution of 1∶50 (BD biosciences, Switzerland). A rabbit polyclonal anti-furin-antibody (Abcam) was used at a dilution of 1∶1500. All corresponding secondary antibodies were AlexaFluor594-labeled IgGs (1∶300, Invitrogen).

### Targeted doxorubicin-RMS-P3/RR treatment of RMS-bearing mice

Mice with size-matched tumors (∼60 mm^3^) were randomized into five groups and injected once per week for 4 consecutive weeks. The therapeutic groups received weekly either 10 µg doxorubicin or 10 µg equivalent of Dox-RMS-P3/RR conjugate. Control groups received either a mixture of 10 µg free doxorubicin and peptide RMS-P3/RR, an equimolar solution of peptide RMS-P-3/RR alone or PBS alone. Concentrations were confirmed by measuring the absorbance of conjugated doxorubicin at 490 nm. A calibration curve of doxorubicin was used for calculation of equivalent Dox-RMS-P3/RR concentrations. Mice were monitored for weight and tumor growth for 30 days.

## Supporting Information

Figure S1 Phage selection and validation on RMS cells *in vivo* and *in vitro*. (A) Biopanning on RD cells *in vitro* after negative selection on myoblasts (panning I) and (B) fibroblasts (panning II). Enrichment was calculated over non-recombinant phage T7. (C) *In vivo* selection in Rh4 xenografts. Phages from *in vitro* selection before enrichment were pooled and used for *in vivo* selection in Rh4 xenografts. Phages were injected in the tail vein and after 10 min circulation unbound phages were washed by perfusion. Phage binding to tumors and control organs was calculated over non-recombinant phage T7. (D) RMS-P3 and RMS-P6 phage distribution in Rh4 xenografts. Homing of phages in mouse xenografts bearing Rh4 tumors was determined and compared to control organs. Binding was normalized over phage T7 and tissue weight. Mean values of three independent experiments are shown. Numerical values for tumor to organ ratios are indicated above the bars. (E) Quantification of binding of RMS-P3 phage to a panel of tumor cell lines: RMS (RD, Rh30, Rh4), breast tumor (MDA-MB 231), glioblastoma (U87MG), melanoma (A365); and normal myoblasts (SkMC-c) and fibroblast cells (MRC-5). Mean values ± SD of three independent experiments are depicted.
(0.55 MB TIF)

Figure S2 Subcellular localization of FITC-RMS-P3 peptide. (A, B) FITC-RMS-P3/RR cell surface and intracellular localization. RD cells were incubated with 100 nM FITC-RMS-P3 peptide *in vitro* for 30 min at 4°C (A) or 37°C (B), respectively. Fixed cells were stained with undiluted 5.1H11 hybridoma supernatant against the human muscle-specific cell surface antigen N-CAM (red). A secondary rabbit anti-mouse Cy3 antibody (1∶300, Invitrogen) was used for detection. Examination under the confocal laser scan microscope revealed a strong incorporation of the peptide (green) at 37°C (B), but also a distinguishable internalization at 4°C (A). Interestingly, surface localization, as indicated by colocalization with N-CAM staining, was visible only at 4°C suggesting complete internalization of FITC-RMS-P3 peptide at 37°C. Cell nuclei were visualized with DAPI. Nuclei are indicated in grey. Magnification: 63x, scale bars: 20 µm. Laser scan images were acquired on a Leica TCS/SP2 confocal microscope (Leica, Wetzlar, Germany) with a 63x water immersion objective, equipped with both argon ion (488 nm, for fluorescein excitation, PMT window 498-540 nm) and HeNe laser. The 543 nm HeNe laser was used to excite Cy3 (PMT window: 553-600 nm). For the double labeling, images from two different channels were collected separately in every focal plane. Between 5 and 15 sections in the z-axis (xy-mode) or y-axis (xz-mode) were acquired, and maximum projections of the sections encompassing the nucleus were performed. (B, C) Laser confocal microscopy of FITC-RMS-P3 in tumors. FITC-labeled peptide (150 µg) was injected i.v. in mice bearing RD xenografts and allowed to circulate for 10 min. After perfusion, tumors and control organs were removed and peptide distribution was evaluated on cryosections (10 µm) by laser scan confocal microscopy. RD tumor sections show FITC-RMS-P3 (green) and cell nuclei visualized with DAPI (white). FITC-RMS-P3 internalization in tumor cells is visible. Magnification: 63x, scale bars: 20 µm. Laser scan images were acquired on a Leica TCS/SP2 confocal microscope (Leica, Wetzlar, Germany) with a water immersion objective and equipped with both argon ion (PMT window: 488-540 nm) and HeNe laser (PMT window: 553-600 nm). Images were collected separately in every focal plane. 12 sections in the z-axis (xy-mode) or y-axis (xz-mode) were acquired, and maximum projections of the sections encompassing the nucleus were performed.
(3.96 MB TIF)

Figure S3 Phage RMS-P3/RR distribution in RMS xenografts. Phage distribution in mice bearing xenografts was assessed after tail vein injection and circulation. After perfusion, tumors and control organs were removed. Phage were rescued and normalized over weight of tissue. Phage RMS-P3/RR showed a 2-fold increased homing to RD-FUR compared to RD and a 45-fold increased binding compared to RD-PDX tumors. Binding to RD-FUR cells was significantly higher than to RD cells, and both were significantly higher than bindings to any other organ. (One-way ANOVA, Tukey Kramer HSD, p<0.05). Further, binding to RMS-FUR tumor was higher than to muscle (27-fold), brain (17-fold), intestines (6-fold) and kidney (22-fold). Binding of control phage RMS-P3/AA was higher to control organs than to tumors. Indicated are means ± SD of three independent experiments.
(0.81 MB TIF)

## References

[1] De Giovanni C, Landuzzi L, Nicoletti G, Lollini PL, Nanni P (2009). Molecular and cellular biology of rhabdomyosarcoma.. Future Oncol.

[2] Breitfeld PP, Meyer WH (2005). Rhabdomyosarcoma: new windows of opportunity.. Oncologist.

[3] Adams GP, Weiner LM (2005). Monoclonal antibody therapy of cancer.. Nat Biotechnol.

[4] Levene AP, Singh G, Palmieri C (2005). Therapeutic monoclonal antibodies in oncology.. J R Soc Med.

[5] Cortez-Retamozo V, Backmann N, Senter PD, Wernery U, De Baetselier P (2004). Efficient cancer therapy with a nanobody-based conjugate.. Cancer Res.

[6] Stern M, Herrmann R (2005). Overview of monoclonal antibodies in cancer therapy: present and promise.. Crit Rev Oncol Hematol.

[7] Aina OH, Sroka TC, Chen ML, Lam KS (2002). Therapeutic cancer targeting peptides.. Biopolymers.

[8] Enback J, Laakkonen P (2007). Tumour-homing peptides: tools for targeting, imaging and destruction.. Biochem Soc Trans.

[9] Aina O, Liu R, Sutcliffe J, Marik J, Pan C-X (2007). From Combinatorial Chemistry to Cancer-Targeting Peptides.. Molecular pharmaceutics.

[10] Ruoslahti E (2004). Vascular zip codes in angiogenesis and metastasis.. Biochem Soc Trans.

[11] Zurita AJ, Arap W, Pasqualini R (2003). Mapping tumor vascular diversity by screening phage display libraries.. J Control Release.

[12] Bergeron F, Leduc R, Day R (2000). Subtilase-like pro-protein convertases: from molecular specificity to therapeutic applications.. J Mol Endocrinol.

[13] Seidah NG, Chretien M (1999). Proprotein and prohormone convertases: a family of subtilases generating diverse bioactive polypeptides.. Brain Res.

[14] Nakayama K (1997). Furin: a mammalian subtilisin/Kex2p-like endoprotease involved in processing of a wide variety of precursor proteins.. Biochem J.

[15] Thomas G (2002). Furin at the cutting edge: from protein traffic to embryogenesis and disease.. Nat Rev Mol Cell Biol.

[16] Khatib AM, Siegfried G, Chretien M, Metrakos P, Seidah NG (2002). Proprotein convertases in tumor progression and malignancy: novel targets in cancer therapy.. Am J Pathol.

[17] Bassi DE, Fu J, Lopez de Cicco R, Klein-Szanto AJ (2005). Proprotein convertases: “master switches” in the regulation of tumor growth and progression.. Mol Carcinog.

[18] Bassi DE, Mahloogi H, Al-Saleem L, Lopez De Cicco R, Ridge JA (2001). Elevated furin expression in aggressive human head and neck tumors and tumor cell lines.. Mol Carcinog.

[19] Siegfried G, Basak A, Cromlish JA, Benjannet S, Marcinkiewicz J (2003). The secretory proprotein convertases furin, PC5, and PC7 activate VEGF-C to induce tumorigenesis.. J Clin Invest.

[20] McColl BK, Paavonen K, Karnezis T, Harris NC, Davydova N (2007). Proprotein convertases promote processing of VEGF-D, a critical step for binding the angiogenic receptor VEGFR-2.. Faseb J.

[21] Dubois CM, Blanchette F, Laprise MH, Leduc R, Grondin F (2001). Evidence that furin is an authentic transforming growth factor-beta1-converting enzyme.. Am J Pathol.

[22] Khatib AM, Siegfried G, Prat A, Luis J, Chretien M (2001). Inhibition of proprotein convertases is associated with loss of growth and tumorigenicity of HT-29 human colon carcinoma cells: importance of insulin-like growth factor-1 (IGF-1) receptor processing in IGF-1-mediated functions.. J Biol Chem.

[23] Nelsen SM, Christian JL (2009). Site-specific cleavage of BMP4 by furin, PC6 and PC7.. The Journal of biological chemistry.

[24] Stawowy P, Kallisch H, Kilimnik A, Margeta C, Seidah NG (2004). Proprotein convertases regulate insulin-like growth factor 1-induced membrane-type 1 matrix metalloproteinase in VSMCs via endoproteolytic activation of the insulin-like growth factor-1 receptor.. Biochem Biophys Res Commun.

[25] Srour N, Lebel A, McMahon S, Fournier I, Fugere M (2003). TACE/ADAM-17 maturation and activation of sheddase activity require proprotein convertase activity.. FEBS Lett.

[26] Bergeron E, Basak A, Decroly E, Seidah NG (2003). Processing of alpha4 integrin by the proprotein convertases: histidine at position P6 regulates cleavage.. Biochem J.

[27] Varshavsky A, Kessler O, Abramovitch S, Kigel B, Zaffryar S (2008). Semaphorin-3B is an angiogenesis inhibitor that is inactivated by furin-like pro-protein convertases.. Cancer Res.

[28] Scamuffa N, Siegfried G, Bontemps Y, Ma L, Basak A (2008). Selective inhibition of proprotein convertases represses the metastatic potential of human colorectal tumor cells.. The Journal of clinical investigation.

[29] Fugere M, Day R (2005). Cutting back on pro-protein convertases: the latest approaches to pharmacological inhibition.. Trends Pharmacol Sci.

[30] Bassi DE, Lopez De Cicco R, Mahloogi H, Zucker S, Thomas G (2001). Furin inhibition results in absent or decreased invasiveness and tumorigenicity of human cancer cells.. Proc Natl Acad Sci U S A.

[31] Lapierre M, Siegfried G, Scamuffa N, Bontemps Y, Calvo F (2007). Opposing Function of the Proprotein Convertases Furin and PACE4 on Breast Cancer Cells' Malignant Phenotypes: Role of Tissue Inhibitors of Metalloproteinase-1.. Cancer Research.

[32] Sun X, Essalmani R, Seidah NG, Prat A (2009). The proprotein convertase PC5/6 is protective against intestinal tumorigenesis: in vivo mouse model.. Mol Cancer.

[33] Chrétien M, Seidah NG, Basak A, Mbikay M (2008). Proprotein convertases as therapeutic targets.. Expert opinion on therapeutic targets.

[34] Witt H, Hajdin K, Iljin K, Greiner O, Niggli FK (2009). Identification of a rhabdomyosarcoma targeting peptide by phage display with sequence similarities to the tumour lymphatic-homing peptide LyP-1.. Int J Cancer.

[35] Mbikay M, Sirois F, Yao J, Seidah NG, Chretien M (1997). Comparative analysis of expression of the proprotein convertases furin, PACE4, PC1 and PC2 in human lung tumours.. Br J Cancer.

[36] Komada M, Hatsuzawa K, Shibamoto S, Ito F, Nakayama K (1993). Proteolytic processing of the hepatocyte growth factor/scatter factor receptor by furin.. FEBS Lett.

[37] Takahashi S, Nakagawa T, Kasai K, Banno T, Duguay SJ (1995). A second mutant allele of furin in the processing-incompetent cell line, LoVo. Evidence for involvement of the homo B domain in autocatalytic activation.. J Biol Chem.

[38] Jean F, Stella K, Thomas L, Liu G, Xiang Y (1998). alpha1-Antitrypsin Portland, a bioengineered serpin highly selective for furin: application as an antipathogenic agent.. Proc Natl Acad Sci U S A.

[39] Laakkonen P, Porkka K, Hoffman JA, Ruoslahti E (2002). A tumor-homing peptide with a targeting specificity related to lymphatic vessels.. Nat Med.

[40] Joyce JA, Laakkonen P, Bernasconi M, Bergers G, Ruoslahti E (2003). Stage-specific vascular markers revealed by phage display in a mouse model of pancreatic islet tumorigenesis.. Cancer Cell.

[41] Teesalu T, Sugahara KN, Kotamraju VR, Ruoslahti E (2009). C-end rule peptides mediate neuropilin-1-dependent cell, vascular, and tissue penetration.. Proc Natl Acad Sci U S A.

[42] Sugahara KN, Teesalu T, Karmali PP, Kotamraju VR, Agemy L (2009). Tissue-penetrating delivery of compounds and nanoparticles into tumors.. Cancer Cell.

[43] Futaki S (2006). Oligoarginine vectors for intracellular delivery: design and cellular-uptake mechanisms.. Biopolymers.

[44] Nishimura S, Takahashi S, Kamikatahira H, Kuroki Y, Jaalouk DE (2008). Combinatorial targeting of the macropinocytotic pathway in Leukemia and lymphoma cells.. The Journal of biological chemistry.

[45] Decroly E, Wouters S, Di Bello C, Lazure C, Ruysschaert JM (1996). Identification of the paired basic convertases implicated in HIV gp160 processing based on in vitro assays and expression in CD4(+) cell lines.. J Biol Chem.

[46] Kido H, Kamoshita K, Fukutomi A, Katunuma N (1993). Processing protease for gp160 human immunodeficiency virus type I envelope glycoprotein precursor in human T4+ lymphocytes. Purification and characterization.. J Biol Chem.

[47] Laprise MH, Grondin F, Cayer P, McDonald PP, Dubois CM (2002). Furin gene (fur) regulation in differentiating human megakaryoblastic Dami cells: involvement of the proximal GATA recognition motif in the P1 promoter and impact on the maturation of furin substrates.. Blood.

[48] Mercapide J, Lopez De Cicco R, Bassi DE, Castresana JS, Thomas G (2002). Inhibition of furin-mediated processing results in suppression of astrocytoma cell growth and invasiveness.. Clin Cancer Res.

[49] Wick W, Wild-Bode C, Frank B, Weller M (2004). BCL-2-induced glioma cell invasiveness depends on furin-like proteases.. J Neurochem.

[50] Oh P, Li Y, Yu J, Durr E, Krasinska KM (2004). Subtractive proteomic mapping of the endothelial surface in lung and solid tumours for tissue-specific therapy.. Nature.

[51] Fogal V, Zhang L, Krajewski S, Ruoslahti E (2008). Mitochondrial/cell-surface protein p32/gC1qR as a molecular target in tumor cells and tumor stroma.. Cancer Res.

[52] Christian S, Pilch J, Akerman ME, Porkka K, Laakkonen P (2003). Nucleolin expressed at the cell surface is a marker of endothelial cells in angiogenic blood vessels.. J Cell Biol.

[53] Basak A, Khatib AM, Mohottalage D, Basak S, Kolajova M (2009). A novel enediynyl peptide inhibitor of furin that blocks processing of proPDGF-A, B and proVEGF-C.. PLoS One.

[54] Remacle AG, Gawlik K, Golubkov VS, Cadwell GW, Liddington RC (2010). Selective and potent furin inhibitors protect cells from anthrax without significant toxicity.. International Journal of Biochemistry and Cell Biology.

[55] Scholl FA, Betts DR, Niggli FK, Schafer BW (2000). Molecular features of a human rhabdomyosarcoma cell line with spontaneous metastatic progression.. Br J Cancer.

[56] Bassi DE, Mahloogi H, Lopez De Cicco R, Klein-Szanto A (2003). Increased furin activity enhances the malignant phenotype of human head and neck cancer cells.. Am J Pathol.

[57] Hoffman JA, Laakkonen P, Porkka K, Bernasconi M, Ruoslahti E, Clarkson T, Lowman H (2004). In vivo and ex vivo selections using phage-displayed libraries.. Phage Display: A Practical Approach.

[58] Wachtel M, Dettling M, Koscielniak E, Stegmaier S, Treuner J (2004). Gene expression signatures identify rhabdomyosarcoma subtypes and detect a novel t(2;2)(q35;p23) translocation fusing PAX3 to NCOA1.. Cancer Res.

