# Supplementary figures and images for: Furin Targeted Drug Delivery for Treatment of Rhabdomyosarcoma in a Mouse Model
Source: PLoS One. 2010 May 3;5(5):e10445. doi: 10.1371/journal.pone.0010445 (PMC2862740; doi:10.1371/journal.pone.0010445)

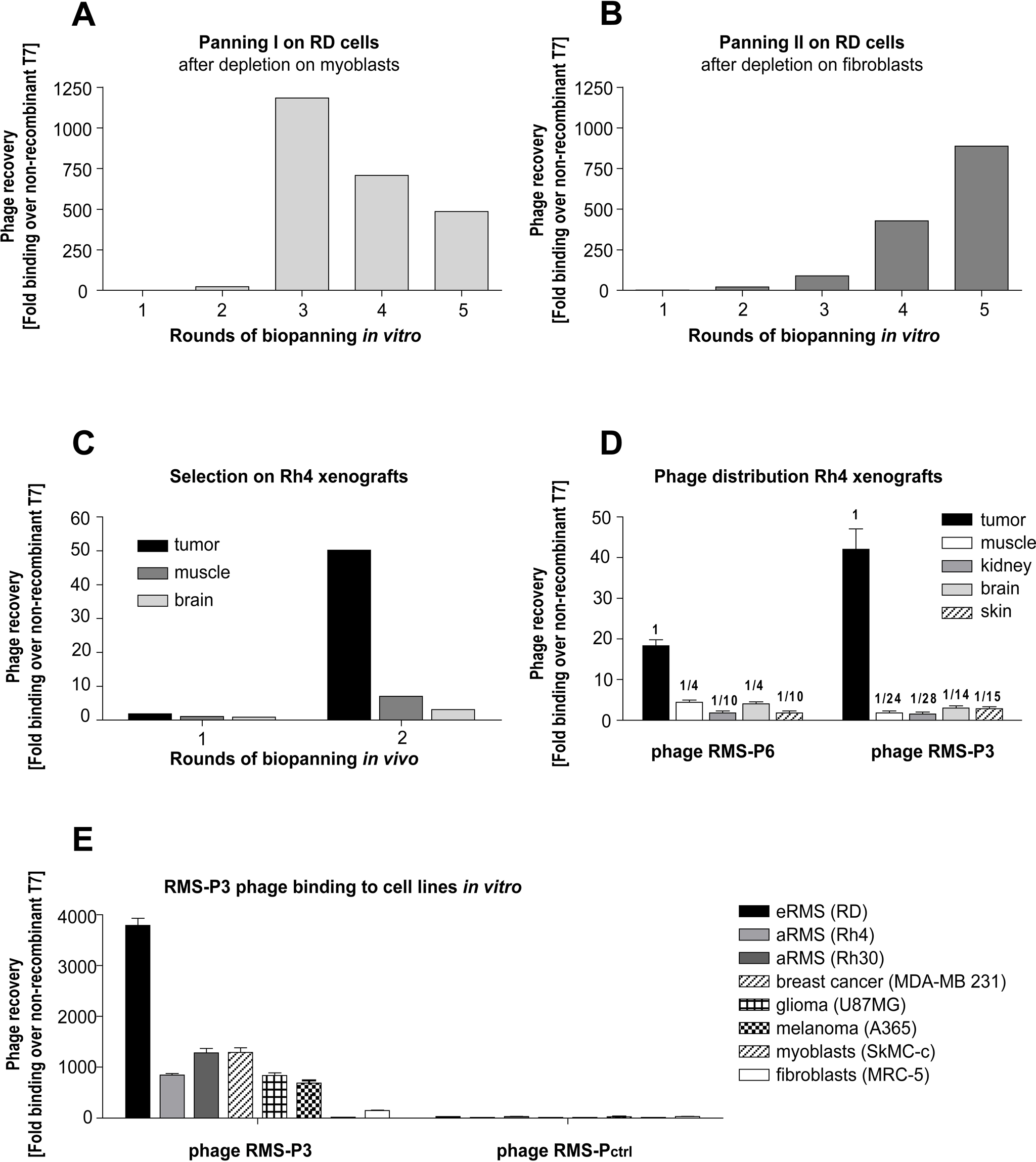

Supplement: Figure S1 — Phage selection and validation on RMS cells in vivo and in vitro. (A) Biopanning on RD cells in vitro after negative selection on myoblasts (panning I) and (B) fibroblasts (panning II). Enrichment was calculated over non-recombinant phage T7. (C) In vivo selection in Rh4 xenografts. Phages from in vitro selection before enrichment were pooled and used for in vivo selection in Rh4 xenografts. Phages were injected in the tail vein and after 10 min circulation unbound phages were washed by perfusion. Phage binding to tumors and control organs was calculated over non-recombinant phage T7. (D) RMS-P3 and RMS-P6 phage distribution in Rh4 xenografts. Homing of phages in mouse xenografts bearing Rh4 tumors was determined and compared to control organs. Binding was normalized over phage T7 and tissue weight. Mean values of three independent experiments are shown. Numerical values for tumor to organ ratios are indicated above the bars. (E) Quantification of binding of RMS-P3 phage to a panel of tumor cell lines: RMS (RD, Rh30, Rh4), breast tumor (MDA-MB 231), glioblastoma (U87MG), melanoma (A365); and normal myoblasts (SkMC-c) and fibroblast cells (MRC-5). Mean values ± SD of three independent experiments are depicted. (0.55 MB TIF) [file pone.0010445.s001.tif]

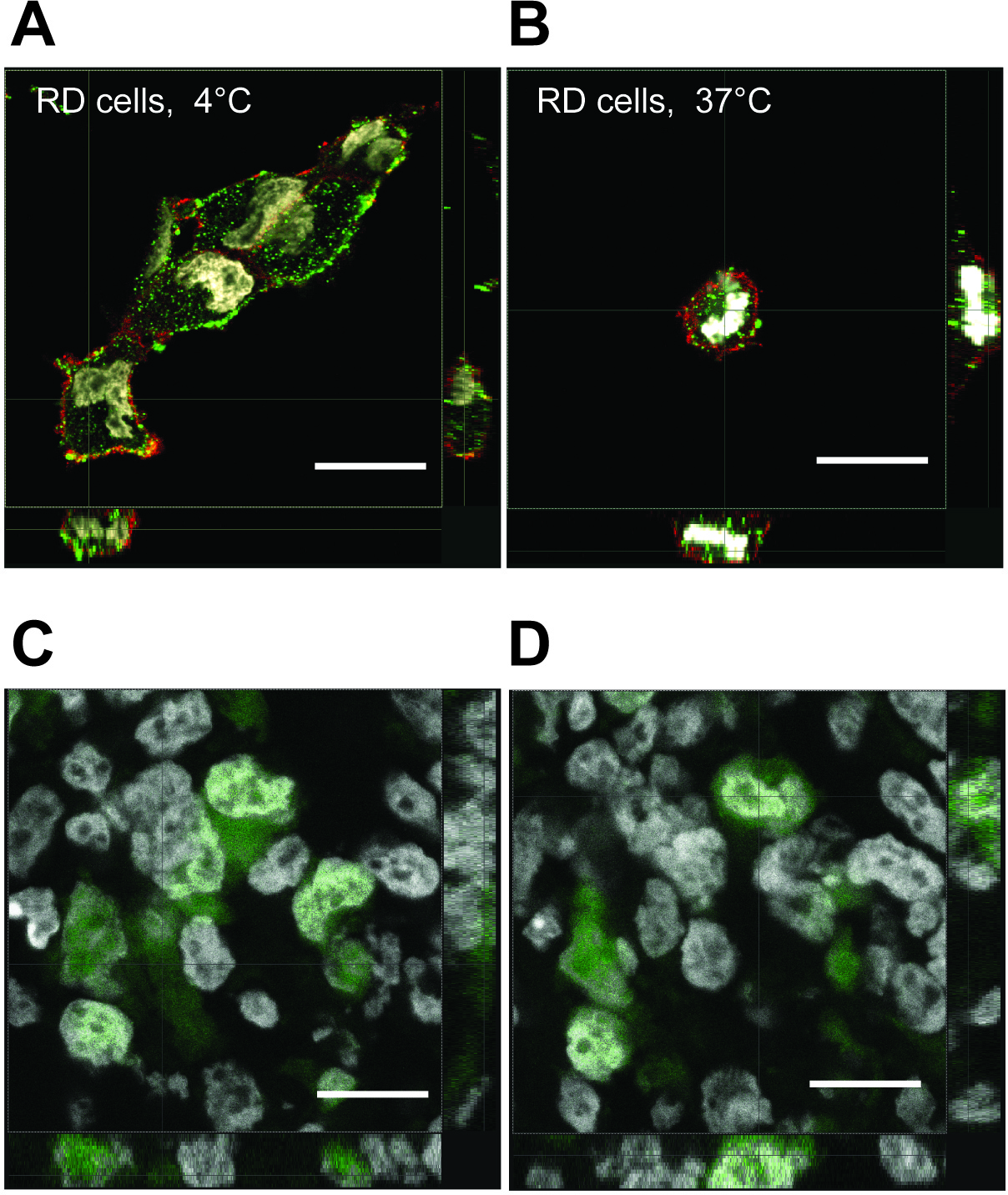

Supplement: Figure S2 — Subcellular localization of FITC-RMS-P3 peptide. (A, B) FITC-RMS-P3/RR cell surface and intracellular localization. RD cells were incubated with 100 nM FITC-RMS-P3 peptide in vitro for 30 min at 4°C (A) or 37°C (B), respectively. Fixed cells were stained with undiluted 5.1H11 hybridoma supernatant against the human muscle-specific cell surface antigen N-CAM (red). A secondary rabbit anti-mouse Cy3 antibody (1∶300, Invitrogen) was used for detection. Examination under the confocal laser scan microscope revealed a strong incorporation of the peptide (green) at 37°C (B), but also a distinguishable internalization at 4°C (A). Interestingly, surface localization, as indicated by colocalization with N-CAM staining, was visible only at 4°C suggesting complete internalization of FITC-RMS-P3 peptide at 37°C. Cell nuclei were visualized with DAPI. Nuclei are indicated in grey. Magnification: 63x, scale bars: 20 µm. Laser scan images were acquired on a Leica TCS/SP2 confocal microscope (Leica, Wetzlar, Germany) with a 63x water immersion objective, equipped with both argon ion (488 nm, for fluorescein excitation, PMT window 498-540 nm) and HeNe laser. The 543 nm HeNe laser was used to excite Cy3 (PMT window: 553-600 nm). For the double labeling, images from two different channels were collected separately in every focal plane. Between 5 and 15 sections in the z-axis (xy-mode) or y-axis (xz-mode) were acquired, and maximum projections of the sections encompassing the nucleus were performed. (B, C) Laser confocal microscopy of FITC-RMS-P3 in tumors. FITC-labeled peptide (150 µg) was injected i.v. in mice bearing RD xenografts and allowed to circulate for 10 min. After perfusion, tumors and control organs were removed and peptide distribution was evaluated on cryosections (10 µm) by laser scan confocal microscopy. RD tumor sections show FITC-RMS-P3 (green) and cell nuclei visualized with DAPI (white). FITC-RMS-P3 internalization in tumor cells is visible. Magnification: 63x, scal [file pone.0010445.s002.tif]

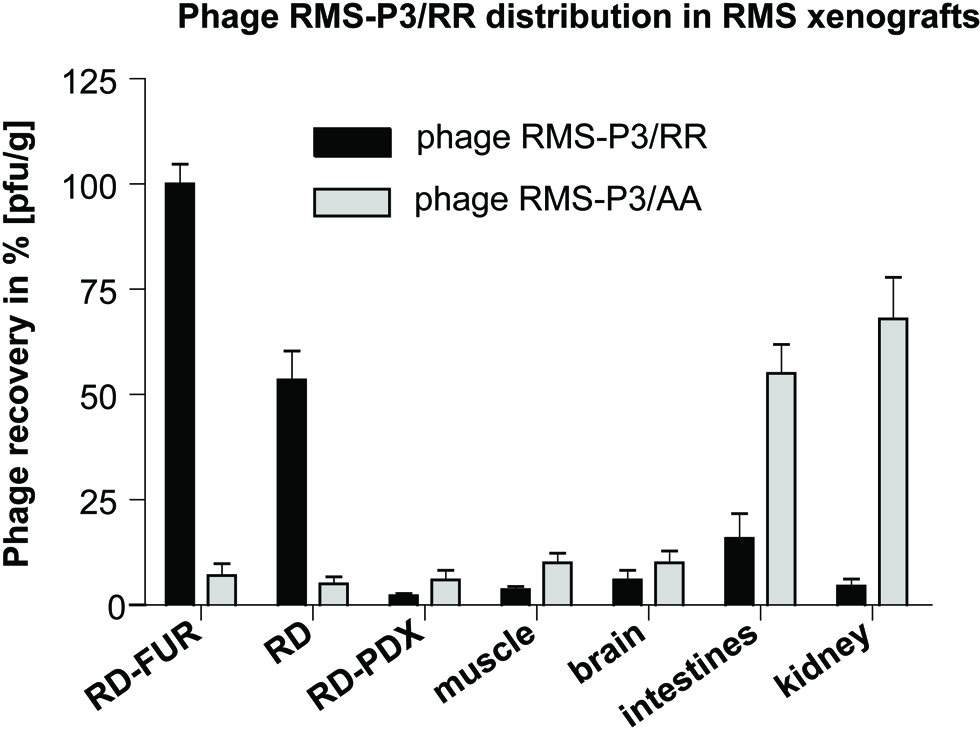

Supplement: Figure S3 — Phage RMS-P3/RR distribution in RMS xenografts. Phage distribution in mice bearing xenografts was assessed after tail vein injection and circulation. After perfusion, tumors and control organs were removed. Phage were rescued and normalized over weight of tissue. Phage RMS-P3/RR showed a 2-fold increased homing to RD-FUR compared to RD and a 45-fold increased binding compared to RD-PDX tumors. Binding to RD-FUR cells was significantly higher than to RD cells, and both were significantly higher than bindings to any other organ. (One-way ANOVA, Tukey Kramer HSD, p<0.05). Further, binding to RMS-FUR tumor was higher than to muscle (27-fold), brain (17-fold), intestines (6-fold) and kidney (22-fold). Binding of control phage RMS-P3/AA was higher to control organs than to tumors. Indicated are means ± SD of three independent experiments. (0.81 MB TIF) [file pone.0010445.s003.tif]
